# The Lost Crown: A Case of an Aspirated Tooth Crown Causing Post-Obstructive Pneumonia

**DOI:** 10.1155/2023/4863886

**Published:** 2023-03-08

**Authors:** Gabriella Primera, Jessika Matta, Louis Eubank, Puncho Gurung

**Affiliations:** ^1^Department of Internal Medicine, University of Massachusetts Chan Medical School, Baystate Medical Center, Springfield, MA, USA; ^2^Department of Pulmonary & Critical Care, Baystate Medical Center, Springfield, MA, USA

## Abstract

Non-asphyxiating foreign body aspiration (FBA) is an uncommon occurrence in adults, but it can lead to serious complications and sequelae. Diagnosis of FBA can be difficult as symptoms can mimic other respiratory diseases and the majority of foreign bodies are not visible on chest X-ray. We report a case of an older male who presented with respiratory failure secondary to pneumonia after aspiration of a dental crown. The patient improved after antibiotic therapy and removal of the foreign body by bronchoscopy. Our case is unusual because the diagnosis was delayed after the aspiration event because the patient was asymptomatic before presenting with pneumonia two years later. This case emphasizes the importance of early recognition and management of possible aspiration events to prevent life-threatening sequelae.

## 1. Introduction

Foreign body aspiration (FBA) can be a serious and sometimes fatal medical condition that needs prompt identification and early intervention to prevent serious complications, such as respiratory failure, airway edema, infection, and pneumothorax. We report a case of a 93-year-old male who was found to have post-obstructive pneumonia secondary to an inhaled tooth two years prior during a dental procedure. Aspiration of a tooth is frequently seen with maxillofacial injuries or dental procedures and is most common in children, elderly patients, and those at high risk of aspiration, including individuals with altered mentation and neurologic disorders [1, 2]. However, aspiration of a tooth remains an uncommon occurrence and accounts for approximately 0.4% of all cases of FBA [2].

## 2. Case Presentation

**Figure 1:**
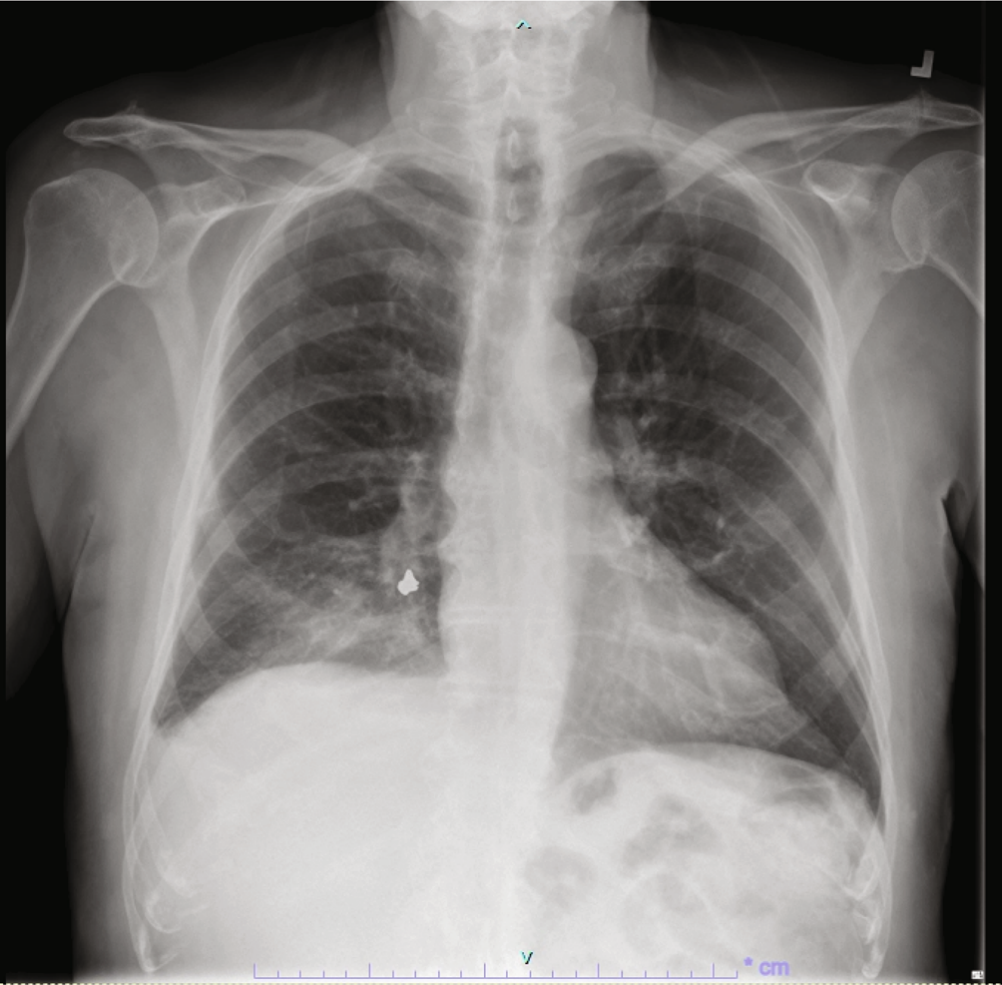
Anterior/posterior chest X-ray demonstrating radiopaque foreign body.

**Figure 2:**
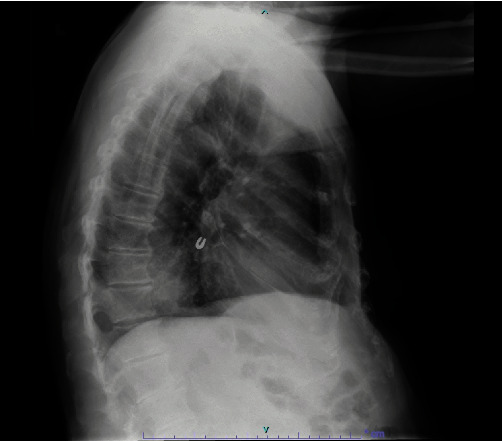
Lateral chest X-ray demonstrating radiopaque foreign body.

**Figure 3:**
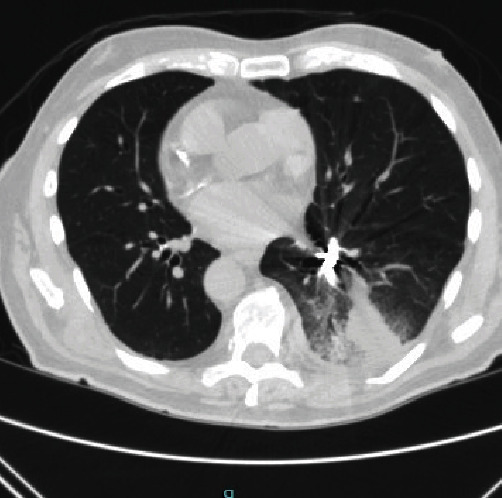
CT scan chest axial section (lung window) with a metallic object in RLL.

**Figure 4:**
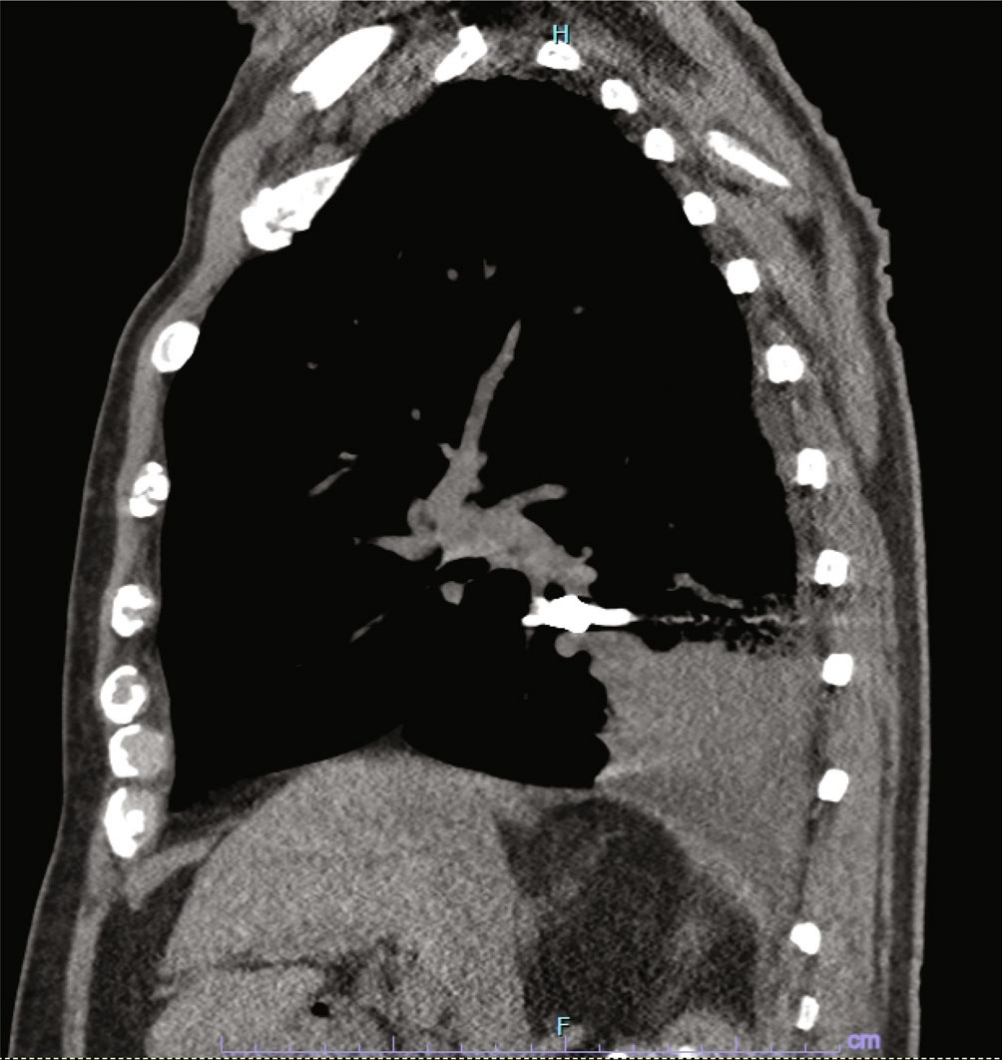
CT chest (sagittal view) with a metallic object and RLL consolidation.

**Figure 5:**
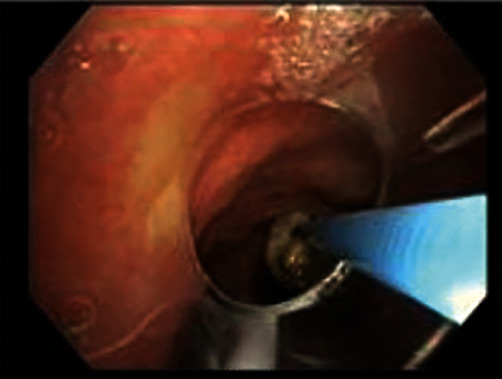
Bronchoscopic view of an aspirated crown at the end of forceps.

A 93-year-old male with coronary artery disease and acid reflux presented to the emergency department from his skilled nursing facility for 4 days of gradually worsening weakness, dry cough, and shortness of breath refractory to outpatient management of presumptive community-acquired pneumonia. The patient was diagnosed with right lower lobe (RLL) pneumonia by the facility's physician four days prior and was prescribed antibiotics. He completed 2 days of antibiotics before presenting to the hospital for continued shortness of breath and intermittent fever. Physical exam was significant for an elderly male in mild respiratory distress, visibly dyspneic with exertion and speaking. He had RLL rhonchi on auscultation, but was not hypoxic on room air. The patient was hemodynamically stable. Laboratory tests were notable for no leukocytosis and mild normocytic anemia. A chest X-ray demonstrated a RLL consolidation with a radioopaque, horseshoe-shaped density in the right infrahilar region consistent with a foreign body (Figures 1 and 2). The patient was treated with ceftriaxone and azithromycin. Upon further history, the patient recalled undergoing crown removal two years ago, where the dentist informed him two pieces came out relatively easy but the third piece was lost during extraction. During the procedure, the patient denied any immediate symptoms including coughing. The patient was asymptomatic following the event and it was assumed that he may have swallowed the lost piece of the crown. No chest imaging was done at the time. After pulmonology consultation, computerized tomography (CT) chest scan without contrast was obtained to characterize the location of the foreign body and surrounding edema or scarring (Figures 3 and 4). This demonstrated a metallic foreign body projecting within a subsegmental bronchus in the posterior RLL suggestive of an aspirated dental crown and distal consolidation consistent with post-obstructive pneumonia. The patient subsequently underwent bronchoscopy to remove the foreign body. Bronchoscopy showed a gold crown in the RLL with surrounding purulent mucus material, but no granulation tissue (Figure 5). The tooth was extracted using forceps without complications. He was discharged the following day on amoxicillin/clavulanate to complete his treatment for post-obstructive pneumonia.

## 3. Discussion

Non-asphyxiating FBA in adults, unlike in children, is an uncommon phenomenon. Patients can present with a broad array of symptoms ranging from cough (81% of the cases), hemoptysis, wheezing, and dyspnea to being completely asymptomatic. These symptoms can be easily misdiagnosed as more common diseases, such as bronchitis or asthma, given their similar presentation [3]. This case was unique in that the patient was asymptomatic post-aspiration and, hence, made the dentist believe he must have swallowed the foreign body instead of aspiration. Up to 80% of foreign bodies are not visible on a chest X-ray, and CT scan of the chest may be valuable in identifying small, aspirated objects [4]. The workup typically includes assessing and securing an airway, followed by a chest X-ray and flexible or rigid bronchoscopy for definite diagnosis and extraction of the non-asphyxiating foreign body. It was previously reported aspiration and long-standing retention of organic foreign bodies are associated with more complications including more severe mucosal inflammation, bronchiectasis, scarring, and granulation tissue compared with inorganic foreign bodies [5]. However, a recent study found no difference in injury severity between organic and inorganic foreign bodies, but additional studies are warranted [6]. This patient's bronchoscopy showed minimal amount of pus around the foreign body within the subsegmental bronchus, but no evidence of inflammation with scarring or granulation tissue, which we suspect is related to the aspiration of an inorganic and inert material. A detailed history including assessment for poor dental hygiene, seizure episodes, substance use disorders, and swallowing of unusual objects, followed by a thorough physical exam of the oral cavity is essential if the diagnosis is uncertain. Our case is unusual in that the diagnosis of tooth aspiration was delayed until two years after the event when he presented with pneumonia. Due to the general asymptomatic time course of aspiration events, sequelae, such as post-obstructive pneumonia secondary to FBA, lung abscess, granulation tissue, and bronchiectasis, can be seen in a significant number of patients with late presentation [7].

## Data Availability

Data supporting this research article are available from the corresponding author or first author on reasonable request.
